# Crystal structure of (*E*)-*N*-{2-[2-(2-chloro­benzyl­idene)hydrazin-1-yl]-2-oxoeth­yl}-4-methyl­benzamide monohydrate

**DOI:** 10.1107/S2056989015011147

**Published:** 2015-06-17

**Authors:** H. Purandara, Sabine Foro, B. Thimme Gowda

**Affiliations:** a Department of Chemistry, Mangalore University, Mangalagangotri 574 199, Mangalore, India; b Institute of Materials Science, Darmstadt University of Technology, Alarich Weiss Strasse 2, D-64287 Darmstadt, Germany; c Bangalore University, Jnanabharati, Bangalore 560 056, India

**Keywords:** crystal structure, glycinyl hydrazone, monohydrate, hydrogen bonding

## Abstract

The title compound, C_17_H_16_ClN_3_O_2_·H_2_O, an acyl­hydrazone derivative, contains a glycine moiety and two substituted benzene rings on either end of the chain. It crystallized as a monohydrate. The mol­ecules adopt an *E* conformation with respect to the C=N double bond, as indicated by the N—N=C—C torsion angle of 179.38 (14)°. The mol­ecule is twisted in such a way that the almost planar C_ar_—C(=O)—N(H)—C(H_2_) and C(H_2_)—C(=O)N(H)—N=C—C_ar_ [r.m.s deviations = 0.009 and 0.025 Å, respectively] segments are inclined to on another by 77.36 (8)°, while the benzene rings are normal to one another, making a dihedral angle of 89.69 (9)°. In the crystal, the water mol­ecule links three mol­ecules through two O—H⋯O and one N—H⋯O hydrogen bonds. The mol­ecules are linked *via* pairs of N—H⋯O hydrogen bonds, forming inversion dimers with an *R*_2_^2^(14) ring motif. The dimers are linked by O—H⋯O hydrogen bonds, involving two mol­ecules of water, forming chains along [100], enclosing *R*_2_^2^(14) and *R*_2_^2^(18) ring motifs. The chains are linked through C—H⋯O inter­actions, forming sheets parallel to (010). Within the sheets, there are C—H⋯π and parallel slipped π–π stacking inter­actions present [inter-centroid distance = 3.6458 (12) Å].

## Abstract 2

The title compound is twisted in such a way that the almost planar [C_ar_—C(=O)—N(H)—C(H_2_] and [C(H_2_)—C(=O)N(H)—N=C(H)—C_ar_] segments are inclined to on another by 77.36 (8)°, while the benzene rings are inclined to one another by 89.69 (9)°. In the crystal, mol­ecules are linked *via* pairs of N—H⋯O hydrogen bonds, forming inversion dimers which are linked by O—H⋯O hydrogen bonds, involving the crystal water mol­ecule, forming chains propagating along the *a-*axis direction.

## Chemical context   

*N*-Acyl­hydrazones have been reported to be promising in terms of their future potential as anti­bacterial drugs (Osorio *et al.*, 2012 ▸). These predictions have provided a therapeutic pathway to develop new effective biologically active Schiff-base derivatives. *N*-Acyl­hydrazones may exist as *Z/E* geom­etrical isomers about the C=N double bond and as *syn/anti* amide conformers (Palla *et al.*, 1986 ▸). The carbonyl group in the acyl­hydrazone provides the possibility for electron delocal­ization within the hydrazone moiety. The anti-TNF-α activity of glycinyl-hydrazone derivatives indicate that differences in the hydro­phobicity of the imine-attached framework plays an important role. The study of conformational isomers of the amide unit of an *N*-methyl *N*-acyl­hydrazone derivative suggested that the amino spacer does not participate as a hydrogen-bond donor in the stabilization of the conformational isomers in solution (Lacerda *et al.*, 2012 ▸). 
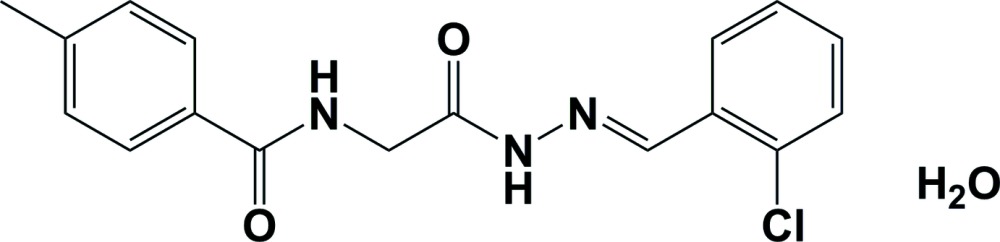


Prompted by the biological and structural importance of Schiff bases, as part of our structural studies (Gowda *et al.*, 2000 ▸; Rodrigues *et al.*, 2011 ▸; Jyothi & Gowda, 2004 ▸; Usha & Gowda, 2006 ▸; Purandara *et al.*, 2015 ▸), we report herein on the synthesis, characterization and crystal structure of the title compound, (I), a new *N*-acyl­hydrazone derivative.

## Structural commentary   

**Figure 1:**
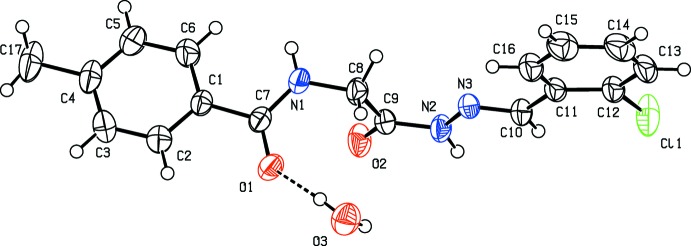
The mol­ecular structure of the title compound, showing the atom labelling. Displacement ellipsoids are drawn at the 50% probability level.

The title compound crystallizes as a monohydrate (Fig. 1 ▸). The conformation of the N—H bond in the amide part is *anti* with respect to both the C=O bonds in the mol­ecule, while the N—H bond in the hydrazone part is *syn* to both the C=O(hydrazone) and the C—H(imine) bonds. The C9—O2 bond length of 1.2251 (19) Å indicates that the mol­ecule exists in the keto form in the solid state, and the C10—N3 bond length of 1.271 (2) Å confirms its significant double-bond character. The C9—N2 and N2—N3 bond distances of 1.351 (2) and 1.3771 (18) Å, respectively, indicate a significant delocalization of the π-electron density over the hydrazone portion of the mol­ecule. Variations in the C—N bond lengths of 1.330 (2), 1.442 (2) and 1.351 (2) Å for C7—N1, C8—N1 and C9—N2, respectively, characterize mobility of the bridge and the integral flexibility of the –C(=O)–NH–CH_2_C(=O)–NH–N=CH– group connecting the two benzene rings. The mol­ecule is twisted at atom C8, the C7—N1—C8—C9 torsion angle being 79.8 (2)°. The hydrazone part of the mol­ecule is almost planar, with C9—N2—N3—C10 and N2—N3—C10—C11 torsion angles of −177.07 (15) and 179.38 (14)°, respectively. Further, the dihedral angle between the almost planar hydrazone segment (O2/N2/N3/C8–C11; maximum deviation of 0.029 (1) Å for atom N2) and the attached benzene ring (C11–C16) is 8.17 (6)°. The two benzene rings (C1–C6 and C11–C16) are orthogonal to each other, making a dihedral angle of 89.69 (9)°. The planar amide segment (O1/N1/C1/C7/C8; r.m.s. deviation = 0.009 Å) is inclined to the attached toluene ring (C1–C6) by 8.06 (9) Å.

## Supra­molecular features   

**Figure 2:**
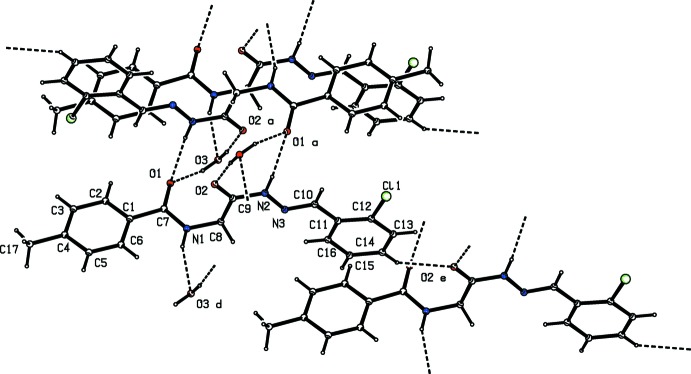
Hydrogen-bonding pattern in the title compound (see Table 1 ▸ for details). [Symmetry codes: (a) −*x* + 1, −*y* + 1, −*z*; (d) *x* + 1, *y*, *z*; (e) *x*, *y*, *z* + 1.]

**Figure 3:**
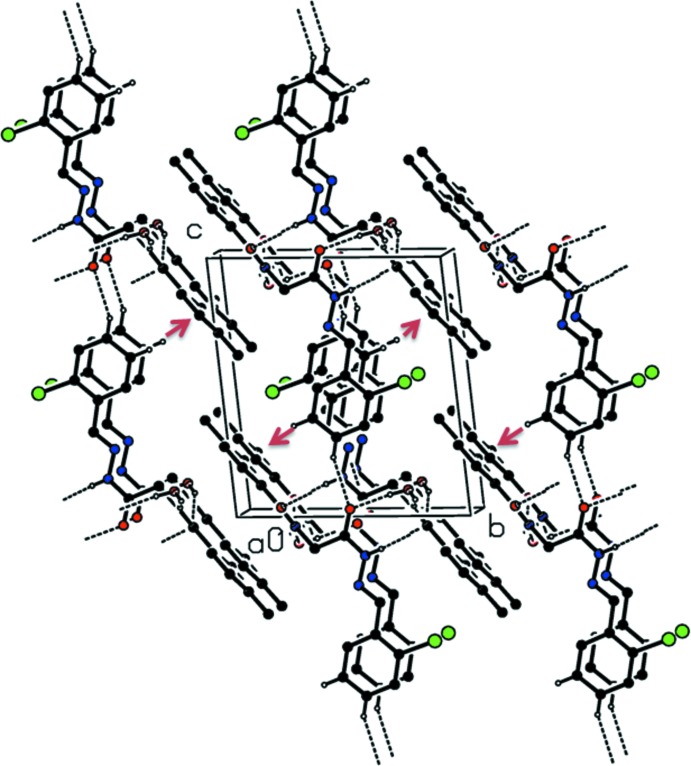
A view along the *a* axis of the crystal packing of the title compound. Hydrogen bonds are shown as dashed lines and C—H⋯π inter­actions are represented as red arrows (see Table 1 ▸ for further details).

In the crystal of (I), the amide carbonyl O-atom, O1, shows bifurcated hydrogen bonding (Table 1 ▸ and Fig. 2 ▸); one with the hydrazide hydrogen atom and the other with one of the hydrogen atoms of the water mol­ecule (O3). The two hydrogen atoms of the water mol­ecule are involved in hydrogen bonding with the O atoms of the amide carbonyl (O3—H31⋯O1) and glycine carbonyl (O3—H32⋯O2) groups of two different mol­ecules of the title compound. The O atom is also involved in hydrogen bonding with the H atom of the carbonyl­amide group of a third symmetry-related mol­ecule (N1—H1*N*⋯O3). A pair of N2—H2*N*⋯O1 inter­molecular hydrogen bonds link the mol­ecules, forming inversion dimers, with an 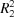
(14) ring motif. The dimers are further linked *via* hydrogen bonds involving the water mol­ecule generating 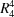
(14) and 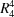
(18) ring motifs. Further, the N2—H2*N*⋯O1 and N1—H1*N*⋯O3 hydrogen bonds between the mol­ecules of the main compound and water mol­ecules translate into 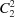
(6) chains along the *a*-axis direction (Table 1 ▸ and Fig. 2 ▸) The chains are linked by a C—H⋯O inter­action, forming sheets parallel to (010). Within the sheets there are C—H⋯π, and parallel slipped π–π stacking inter­actions [*Cg*2⋯*Cg*2^i^ = 3.6458 (12) Å; inter-planar distance = 3.4135 (8) Å, slippage = 1.281 Å; *Cg*2 is the centroid of ring C11–C16; symmetry code: (i) −*x* + 1, −*y* + 1, −*z* + 1] involving inversion-related chloro­benzene rings; see Fig. 3 ▸.

## Database survey   

A search of the Cambridge Structural Database (Version 5.36, May 2015; Groom & Allen, 2014 ▸) for the fragment –NH–CH_2_–C(=O)–NH–N=CH–, yielded only one hit, namely *N*-(2-hy­droxy-1-naphthyl­methyl­ene)-*N*′-(*N*-phenyl­glyc­yl)hydrazine (MEMTOO; Gudasi *et al.*, 2006 ▸). A comparison of the structural details of the title compound, (I), with those of the recently published sulfonyl derivative, (*E*)-*N*-{2-[2-(3-chlorobenzyl­idene)hydrazin­yl]-2-oxoeth­yl}-4-methyl­benzene­sulf­onamide monohydrate (II) (Purandara *et al.*, 2015 ▸), reveals the *trans* orientation of the amide group (C1–C7(=O1)N1) and hydrazone segment (N2–N3=C10–C11) with respect to the glycinyl C8—C9 bond in (I), as is evident from the N1—C8—C9—N2 torsion angle of 173.58 (15)°, in contrast to the *cis* orientation of the sulfonamide and hydrazone segments, with respect to the glycinyl C—C bond, observed in compound (II). In the structure of (I), the benzene ring (C1–C6) is almost coplanar with the amide group [dihedral angle = 8.21 (13)°]. This is in contrast to the L-shaped conformation (bent at the S atom) of the sulfonamide group with respect to the benzene ring in compound (II). The amide carbonyl O atom forms stronger O—H⋯O hydrogen bonds with the water H atoms than the sulfonyl O atom as observed in compound (II), indicating the stronger electron-withdrawing character of the amide group compared to the sulfonamide group.

## Synthesis and crystallization   

Tri­ethyl­amine (0.03 mol) and 4-methyl­benzoyl chloride (0.01 mol) were added to a stirred suspension of glycine ethyl­ester hydro­chloride (0.01 mol) in di­chloro­methane (50 ml) in an ice bath. The reaction mixture was stirred at room temperature for 20 h. After completion of the reaction, 2*N* hydro­chloric acid (80 ml) was added slowly. The organic phase was separated and washed with water (30 ml), dried with anhydrous Na_2_SO_4_ and evaporated to yield the corresponding ester, *N*-(4-methyl­benzo­yl)glycine ethyl ester (*L*1). *L*1 (0.01 mol) was added in small portions to a stirred solution of 99% hydrazine hydrate (10 ml) in 30 ml ethanol. The mixture was refluxed for 6 h. After cooling to room temperature, the resulting precipitate was filtered, washed with cold water and dried to give *N*-(4-methyl­benzo­yl)-glycinyl hydrazide (*L*2). 2-Chloro­benzaldehyde (0.01 mol) and two drops of glacial acetic acid were added to *L*2 (0.01 mol) in anhydrous methanol (30 ml). The reaction mixture was refluxed for 8 h. After cooling, the precipitate was collected by vacuum filtration, washed with cold methanol and dried. It was recrystallized to constant melting point from methanol (479–480 K). Prism-like colourless single crystals of the title compound were grown from a solution in DMF by slow evaporation of the solvent.

The purity of the compound was checked by TLC and characterized by its IR spectrum. The characteristic absorptions observed are 3323.3, 3203.8, 1685.8, 1620.2 and 1566.2 cm^−1^ for the stretching bands of N—H (amide I), N—H (amide II), C=O(hydrazone), C=O(amide) and C=N, respectively. The characteristic ^1^H and ^13^C NMR spectra of the title compound are as follows: ^1^H NMR (400 MHz, DMSO-*d*6, δ p.p.m.): 2.36 (*s*, 3H), 4.01, 4.45 (2*d*, 2H, *J* = 5.8 Hz), 7.25 (*d*, 2H, Ar-H, *J* = 8.0 Hz), 7.33–7.40 (*m*, 2H, Ar-H), 7.42–7.45 (*m*, 1H, Ar-H), 7.81 (*d*, 2H, Ar-H), 7.97–7.99 (*m*, 1H, Ar-H), 8.39, 8.63 (2*s*, 1H), 8.54, 8.76 (2*t*, 1H, *J* = 5.7 Hz), 11.65, 11.73 (2*s*, 1H). ^13^C NMR (400 MHz, DMSO-*d*6, δ p.p.m.): 20.97, 40.74, 42.04, 126.60, 126.83, 127.28, 128.64, 129.66, 130.85, 131.35, 133.10, 139.45, 141.06, 142.70, 165.98, 166.54, 170.48.

## Refinement   

Crystal data, data collection and structure refinement details are summarized in Table 2 ▸. The water H atoms and the NH H atoms were located in a difference Fourier map and refined with distances restraints: O—H = 0.85 (2), N—H = 0.86 (2) Å with *U*_iso_(H) = 1.5*U*_eq_(O) and 1.2*U*_eq_(N). The C-bound H atoms were positioned with idealized geometry and refined as riding atoms: C—H = 0.93–0.97 Å with *U*_iso_(H) = 1.5*U*_eq_(C) for methyl H atoms and 1.2*U*_eq_(C) for other H atoms.

## Supplementary Material

Crystal structure: contains datablock(s) I, global. DOI: 10.1107/S2056989015011147/su5148sup1.cif

Structure factors: contains datablock(s) I. DOI: 10.1107/S2056989015011147/su5148Isup2.hkl

Supporting information file. DOI: 10.1107/S2056989015011147/su5148Isup3.cml

CCDC reference: 1405614

Additional supporting information:  crystallographic information; 3D view; checkCIF report

## Figures and Tables

**Table 1:** Hydrogen-bond geometry (, ) *Cg*1 is the centroid of the toluene ring C1C6.

*D*H*A*	*D*H	H*A*	*D* *A*	*D*H*A*
O3H31O1	0.84(2)	2.13(2)	2.897(2)	152(3)
O3H32O2^i^	0.86(2)	1.92(2)	2.772(2)	174(3)
N1H1*N*O3^ii^	0.84(2)	2.15(2)	2.941(2)	158(2)
N2H2*N*O1^i^	0.87(2)	2.09(2)	2.944(2)	165(2)
C14H14O2^iii^	0.93	2.57	3.404(2)	150
C15H15*Cg*1^iii^	0.93	2.89	3.793(2)	165

Symmetry codes: (i) 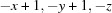; (ii) 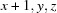; (iii) 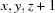.

**Table 2:** Experimental details

Crystal data	
Chemical formula	C_17_H_16_ClN_3_O_2_H_2_O
*M*_r_	347.79
Crystal system, space group	Triclinic, *P* 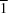
Temperature (K)	293
*a*, *b*, *c* ()	6.9729(7), 10.642(1), 11.879(1)
, , ()	95.049(8), 100.324(9), 102.870(9)
*V* (^3^)	837.88(14)
*Z*	2
Radiation type	Mo *K*
(mm^1^)	0.25
Crystal size (mm)	0.50 0.40 0.32

Data collection	
Diffractometer	Oxford Diffraction Xcalibur with Sapphire CCD detector
Absorption correction	Multi-scan (*CrysAlis RED*; Oxford Diffraction, 2009 ▸)
*T*_min_, *T*_max_	0.886, 0.925
No. of measured, independent and observed [*I* > 2(*I*)] reflections	5538, 3393, 2829
*R*_int_	0.009
(sin /)_max_ (^1^)	0.625

Refinement	
*R*[*F* ^2^ > 2(*F* ^2^)], *wR*(*F* ^2^), *S*	0.039, 0.103, 1.04
No. of reflections	3393
No. of parameters	230
No. of restraints	4
H-atom treatment	H atoms treated by a mixture of independent and constrained refinement
_max_, _min_ (e ^3^)	0.24, 0.33

Computer programs: *CrysAlis CCD* and *CrysAlis RED* (Oxford Diffraction, 2009 ▸), *SHELXS97* and *SHELXL97* (Sheldrick, 2008 ▸) and *PLATON* (Spek, 2009 ▸).

## supplementary crystallographic information

### Crystal data

**Table:**

C_17_H_16_ClN_3_O_2_·H_2_O	*Z* = 2
*M**_r_* = 347.79	*F*(000) = 364
Triclinic, *P*1	*D*_x_ = 1.379 Mg m^−^^3^
Hall symbol: -P 1	Mo *K*α radiation, λ = 0.71073 Å
*a* = 6.9729 (7) Å	Cell parameters from 3287 reflections
*b* = 10.642 (1) Å	θ = 3.1–27.7°
*c* = 11.879 (1) Å	µ = 0.25 mm^−^^1^
α = 95.049 (8)°	*T* = 293 K
β = 100.324 (9)°	Prism, colourless
γ = 102.870 (9)°	0.50 × 0.40 × 0.32 mm
*V* = 837.88 (14) Å^3^	

### Data collection

**Table:**

Oxford Diffraction Xcalibur single crystal X-ray diffractometer with a Sapphire CCD detector	3393 independent reflections
Radiation source: fine-focus sealed tube	2829 reflections with *I* > 2σ(*I*)
Graphite monochromator	*R*_int_ = 0.009
Rotation method data acquisition using ω scans	θ_max_ = 26.4°, θ_min_ = 3.1°
Absorption correction: multi-scan (*CrysAlis RED*; Oxford Diffraction, 2009)	*h* = −7→8
*T*_min_ = 0.886, *T*_max_ = 0.925	*k* = −12→13
5538 measured reflections	*l* = −14→11

### Refinement

**Table:**

Refinement on *F*^2^	Primary atom site location: structure-invariant direct methods
Least-squares matrix: full	Secondary atom site location: difference Fourier map
*R*[*F*^2^ > 2σ(*F*^2^)] = 0.039	Hydrogen site location: inferred from neighbouring sites
*wR*(*F*^2^) = 0.103	H atoms treated by a mixture of independent and constrained refinement
*S* = 1.04	*w* = 1/[σ^2^(*F*_o_^2^) + (0.0396*P*)^2^ + 0.4048*P*] where *P* = (*F*_o_^2^ + 2*F*_c_^2^)/3
3393 reflections	(Δ/σ)_max_ < 0.001
230 parameters	Δρ_max_ = 0.24 e Å^−^^3^
4 restraints	Δρ_min_ = −0.33 e Å^−^^3^

### Special details

**Table:**

Geometry. All e.s.d.'s (except the e.s.d. in the dihedral angle between two l.s. planes) are estimated using the full covariance matrix. The cell e.s.d.'s are taken into account individually in the estimation of e.s.d.'s in distances, angles and torsion angles; correlations between e.s.d.'s in cell parameters are only used when they are defined by crystal symmetry. An approximate (isotropic) treatment of cell e.s.d.'s is used for estimating e.s.d.'s involving l.s. planes.
Refinement. Refinement of *F*^2^ against ALL reflections. The weighted *R*-factor *wR* and goodness of fit *S* are based on *F*^2^, conventional *R*-factors *R* are based on *F*, with *F* set to zero for negative *F*^2^. The threshold expression of *F*^2^ > σ(*F*^2^) is used only for calculating *R*-factors(gt) *etc*. and is not relevant to the choice of reflections for refinement. *R*-factors based on *F*^2^ are statistically about twice as large as those based on *F*, and *R*-factors based on ALL data will be even larger.

### Fractional atomic coordinates and isotropic or equivalent isotropic displacement parameters (Å^2^)

**Table:**

	*x*	*y*	*z*	*U*_iso_*/*U*_eq_	
Cl1	0.60321 (11)	0.19118 (5)	0.47068 (5)	0.0724 (2)	
O1	0.62994 (17)	0.78208 (12)	−0.04768 (11)	0.0451 (3)	
O2	0.7257 (2)	0.49520 (12)	−0.03656 (10)	0.0499 (3)	
N1	0.9290 (2)	0.75016 (14)	0.03466 (12)	0.0417 (3)	
H1N	1.054 (2)	0.766 (2)	0.0404 (17)	0.050*	
N2	0.6958 (2)	0.45136 (14)	0.14178 (11)	0.0395 (3)	
H2N	0.617 (3)	0.3748 (16)	0.1142 (16)	0.047*	
N3	0.7330 (2)	0.49621 (14)	0.25783 (11)	0.0365 (3)	
C1	0.9132 (2)	0.87432 (15)	−0.12558 (13)	0.0340 (3)	
C2	0.8034 (3)	0.93929 (18)	−0.19759 (16)	0.0470 (4)	
H2	0.6703	0.9352	−0.1934	0.056*	
C3	0.8883 (3)	1.0105 (2)	−0.27602 (17)	0.0543 (5)	
H3	0.8112	1.0537	−0.3235	0.065*	
C4	1.0838 (3)	1.01864 (17)	−0.28502 (15)	0.0471 (4)	
C5	1.1929 (3)	0.9537 (2)	−0.21334 (17)	0.0551 (5)	
H5	1.3259	0.9581	−0.2179	0.066*	
C6	1.1104 (3)	0.8822 (2)	−0.13474 (17)	0.0503 (5)	
H6	1.1879	0.8390	−0.0876	0.060*	
C7	0.8130 (2)	0.79820 (15)	−0.04311 (13)	0.0349 (3)	
C8	0.8517 (3)	0.67330 (17)	0.11810 (14)	0.0418 (4)	
H8A	0.7536	0.7113	0.1479	0.050*	
H8B	0.9608	0.6755	0.1822	0.050*	
C9	0.7542 (2)	0.53317 (16)	0.06684 (13)	0.0365 (4)	
C10	0.6807 (2)	0.41135 (17)	0.32262 (14)	0.0383 (4)	
H10	0.6205	0.3256	0.2906	0.046*	
C11	0.7154 (2)	0.44844 (17)	0.44785 (13)	0.0365 (4)	
C12	0.6834 (3)	0.35608 (18)	0.52341 (15)	0.0426 (4)	
C13	0.7134 (3)	0.3916 (2)	0.64139 (15)	0.0517 (5)	
H13	0.6894	0.3284	0.6899	0.062*	
C14	0.7787 (3)	0.5207 (2)	0.68642 (16)	0.0558 (5)	
H14	0.7984	0.5451	0.7656	0.067*	
C15	0.8151 (3)	0.6143 (2)	0.61429 (16)	0.0539 (5)	
H15	0.8613	0.7016	0.6448	0.065*	
C16	0.7828 (3)	0.57766 (19)	0.49659 (15)	0.0453 (4)	
H16	0.8069	0.6415	0.4487	0.054*	
C17	1.1791 (4)	1.0976 (2)	−0.36898 (19)	0.0688 (6)	
H17A	1.0858	1.1420	−0.4073	0.103*	
H17B	1.2140	1.0409	−0.4252	0.103*	
H17C	1.2981	1.1602	−0.3279	0.103*	
O3	0.3605 (2)	0.76894 (15)	0.11275 (13)	0.0555 (4)	
H31	0.452 (3)	0.799 (3)	0.078 (2)	0.083*	
H32	0.340 (4)	0.6869 (17)	0.094 (2)	0.083*	

### Atomic displacement parameters (Å^2^)

**Table:**

	*U*^11^	*U*^22^	*U*^33^	*U*^12^	*U*^13^	*U*^23^
Cl1	0.1083 (5)	0.0530 (3)	0.0572 (3)	0.0101 (3)	0.0233 (3)	0.0258 (2)
O1	0.0388 (6)	0.0496 (7)	0.0445 (7)	0.0007 (5)	0.0129 (5)	0.0100 (5)
O2	0.0698 (9)	0.0503 (7)	0.0277 (6)	0.0064 (6)	0.0134 (6)	0.0093 (5)
N1	0.0383 (7)	0.0470 (8)	0.0364 (7)	−0.0009 (6)	0.0082 (6)	0.0159 (6)
N2	0.0474 (8)	0.0396 (8)	0.0278 (7)	0.0010 (6)	0.0081 (6)	0.0094 (6)
N3	0.0372 (7)	0.0458 (8)	0.0270 (6)	0.0077 (6)	0.0077 (5)	0.0115 (6)
C1	0.0395 (8)	0.0314 (8)	0.0285 (7)	0.0016 (6)	0.0088 (6)	0.0038 (6)
C2	0.0442 (10)	0.0510 (10)	0.0485 (10)	0.0116 (8)	0.0115 (8)	0.0165 (8)
C3	0.0641 (12)	0.0526 (11)	0.0495 (11)	0.0152 (9)	0.0112 (9)	0.0241 (9)
C4	0.0656 (12)	0.0367 (9)	0.0349 (9)	−0.0020 (8)	0.0169 (8)	0.0061 (7)
C5	0.0463 (10)	0.0709 (13)	0.0527 (11)	0.0093 (9)	0.0227 (9)	0.0204 (10)
C6	0.0454 (10)	0.0651 (12)	0.0476 (10)	0.0167 (9)	0.0155 (8)	0.0250 (9)
C7	0.0384 (8)	0.0322 (8)	0.0303 (8)	−0.0005 (6)	0.0090 (6)	0.0028 (6)
C8	0.0467 (9)	0.0454 (9)	0.0290 (8)	0.0003 (7)	0.0072 (7)	0.0117 (7)
C9	0.0379 (8)	0.0433 (9)	0.0288 (8)	0.0075 (7)	0.0077 (6)	0.0115 (7)
C10	0.0424 (9)	0.0427 (9)	0.0322 (8)	0.0093 (7)	0.0112 (7)	0.0126 (7)
C11	0.0328 (8)	0.0508 (10)	0.0308 (8)	0.0137 (7)	0.0102 (6)	0.0153 (7)
C12	0.0396 (9)	0.0558 (10)	0.0377 (9)	0.0141 (8)	0.0123 (7)	0.0196 (8)
C13	0.0470 (10)	0.0817 (15)	0.0340 (9)	0.0208 (10)	0.0120 (8)	0.0276 (9)
C14	0.0490 (11)	0.0920 (16)	0.0288 (9)	0.0226 (10)	0.0071 (8)	0.0091 (9)
C15	0.0540 (11)	0.0652 (13)	0.0408 (10)	0.0157 (9)	0.0067 (8)	0.0015 (9)
C16	0.0478 (10)	0.0527 (11)	0.0382 (9)	0.0135 (8)	0.0112 (7)	0.0140 (8)
C17	0.0966 (17)	0.0565 (12)	0.0513 (12)	−0.0029 (12)	0.0318 (12)	0.0176 (10)
O3	0.0576 (8)	0.0586 (8)	0.0554 (8)	0.0145 (7)	0.0223 (7)	0.0109 (7)

### Geometric parameters (Å, º)

**Table:**

Cl1—C12	1.740 (2)	C6—H6	0.9300
O1—C7	1.240 (2)	C8—C9	1.516 (2)
O2—C9	1.2251 (19)	C8—H8A	0.9700
N1—C7	1.330 (2)	C8—H8B	0.9700
N1—C8	1.442 (2)	C10—C11	1.467 (2)
N1—H1N	0.842 (15)	C10—H10	0.9300
N2—C9	1.351 (2)	C11—C16	1.386 (3)
N2—N3	1.3771 (18)	C11—C12	1.397 (2)
N2—H2N	0.873 (15)	C12—C13	1.385 (3)
N3—C10	1.271 (2)	C13—C14	1.373 (3)
C1—C2	1.379 (2)	C13—H13	0.9300
C1—C6	1.383 (2)	C14—C15	1.381 (3)
C1—C7	1.496 (2)	C14—H14	0.9300
C2—C3	1.384 (3)	C15—C16	1.382 (3)
C2—H2	0.9300	C15—H15	0.9300
C3—C4	1.371 (3)	C16—H16	0.9300
C3—H3	0.9300	C17—H17A	0.9600
C4—C5	1.373 (3)	C17—H17B	0.9600
C4—C17	1.510 (2)	C17—H17C	0.9600
C5—C6	1.380 (2)	O3—H31	0.840 (17)
C5—H5	0.9300	O3—H32	0.856 (17)
			
C7—N1—C8	122.85 (15)	H8A—C8—H8B	107.9
C7—N1—H1N	121.7 (14)	O2—C9—N2	121.16 (16)
C8—N1—H1N	115.4 (14)	O2—C9—C8	122.74 (14)
C9—N2—N3	119.90 (14)	N2—C9—C8	116.08 (14)
C9—N2—H2N	118.6 (13)	N3—C10—C11	120.13 (16)
N3—N2—H2N	120.7 (13)	N3—C10—H10	119.9
C10—N3—N2	115.65 (14)	C11—C10—H10	119.9
C2—C1—C6	117.83 (15)	C16—C11—C12	116.92 (16)
C2—C1—C7	118.58 (15)	C16—C11—C10	121.14 (15)
C6—C1—C7	123.59 (15)	C12—C11—C10	121.94 (16)
C1—C2—C3	121.00 (17)	C13—C12—C11	121.76 (18)
C1—C2—H2	119.5	C13—C12—Cl1	117.92 (14)
C3—C2—H2	119.5	C11—C12—Cl1	120.32 (14)
C4—C3—C2	121.22 (18)	C14—C13—C12	119.63 (17)
C4—C3—H3	119.4	C14—C13—H13	120.2
C2—C3—H3	119.4	C12—C13—H13	120.2
C3—C4—C5	117.71 (16)	C13—C14—C15	120.05 (17)
C3—C4—C17	121.75 (19)	C13—C14—H14	120.0
C5—C4—C17	120.53 (19)	C15—C14—H14	120.0
C4—C5—C6	121.76 (18)	C14—C15—C16	119.8 (2)
C4—C5—H5	119.1	C14—C15—H15	120.1
C6—C5—H5	119.1	C16—C15—H15	120.1
C5—C6—C1	120.49 (17)	C15—C16—C11	121.87 (17)
C5—C6—H6	119.8	C15—C16—H16	119.1
C1—C6—H6	119.8	C11—C16—H16	119.1
O1—C7—N1	122.19 (14)	C4—C17—H17A	109.5
O1—C7—C1	120.78 (15)	C4—C17—H17B	109.5
N1—C7—C1	117.03 (14)	H17A—C17—H17B	109.5
N1—C8—C9	112.26 (14)	C4—C17—H17C	109.5
N1—C8—H8A	109.2	H17A—C17—H17C	109.5
C9—C8—H8A	109.2	H17B—C17—H17C	109.5
N1—C8—H8B	109.2	H31—O3—H32	102 (3)
C9—C8—H8B	109.2		
			
C9—N2—N3—C10	−177.07 (15)	N3—N2—C9—O2	178.83 (15)
C6—C1—C2—C3	−0.3 (3)	N3—N2—C9—C8	−2.4 (2)
C7—C1—C2—C3	−179.62 (17)	N1—C8—C9—O2	−7.6 (2)
C1—C2—C3—C4	0.2 (3)	N1—C8—C9—N2	173.58 (15)
C2—C3—C4—C5	−0.1 (3)	N2—N3—C10—C11	179.38 (14)
C2—C3—C4—C17	−179.09 (19)	N3—C10—C11—C16	7.7 (2)
C3—C4—C5—C6	0.1 (3)	N3—C10—C11—C12	−171.98 (16)
C17—C4—C5—C6	179.14 (19)	C16—C11—C12—C13	1.3 (2)
C4—C5—C6—C1	−0.3 (3)	C10—C11—C12—C13	−179.00 (16)
C2—C1—C6—C5	0.3 (3)	C16—C11—C12—Cl1	−178.83 (13)
C7—C1—C6—C5	179.62 (17)	C10—C11—C12—Cl1	0.9 (2)
C8—N1—C7—O1	1.4 (3)	C11—C12—C13—C14	−0.8 (3)
C8—N1—C7—C1	−179.01 (15)	Cl1—C12—C13—C14	179.30 (15)
C2—C1—C7—O1	7.7 (2)	C12—C13—C14—C15	−0.3 (3)
C6—C1—C7—O1	−171.59 (17)	C13—C14—C15—C16	0.9 (3)
C2—C1—C7—N1	−171.90 (16)	C14—C15—C16—C11	−0.4 (3)
C6—C1—C7—N1	8.8 (2)	C12—C11—C16—C15	−0.7 (3)
C7—N1—C8—C9	79.8 (2)	C10—C11—C16—C15	179.63 (17)

### Hydrogen-bond geometry (Å, º)

Cg1 is the centroid of the toluene ring C1–C6.

**Table:**

*D*—H···*A*	*D*—H	H···*A*	*D*···*A*	*D*—H···*A*
O3—H31···O1	0.84 (2)	2.13 (2)	2.897 (2)	152 (3)
O3—H32···O2^i^	0.86 (2)	1.92 (2)	2.772 (2)	174 (3)
N1—H1*N*···O3^ii^	0.84 (2)	2.15 (2)	2.941 (2)	158 (2)
N2—H2*N*···O1^i^	0.87 (2)	2.09 (2)	2.944 (2)	165 (2)
C14—H14···O2^iii^	0.93	2.57	3.404 (2)	150
C15—H15···*Cg*1^iii^	0.93	2.89	3.793 (2)	165

Symmetry codes: (i) −*x*+1, −*y*+1, −*z*; (ii) *x*+1, *y*, *z*; (iii) *x*, *y*, *z*+1.
